# Synthesis of Pore-Size-Tunable Porous Silica Particles and Their Effects on Dental Resin Composites

**DOI:** 10.3390/biom13091290

**Published:** 2023-08-24

**Authors:** Hongyan Chen, Jiaxin Luo, Jiawei Yang, Chen Zeng, Xinquan Jiang

**Affiliations:** 1 Shanghai Engineering Research Center of Advanced Dental Technology and Materials, Shanghai 200011, China; b21021@sh9hospital.org.cn (H.C.);; 2 Shanghai Key Laboratory of Stomatology, Shanghai 200011, China; 3 Shanghai Research Institute of Stomatology, Shanghai 200011, China; 4 National Clinical Research Center for Oral Diseases, Shanghai 200011, China; 5 Department of Prosthodontics, Shanghai Ninth People’s Hospital, Shanghai Jiao Tong University School of Medicine, Shanghai 200011, China; 6 College of Stomatology, Shanghai Jiao Tong University, Shanghai 200011, China; 7 State Key Laboratory for Modification of Chemical Fibers and Polymer Materials, College of Material Science and Engineering, Donghua University, Shanghai 201620, China

**Keywords:** dental resin composites, porous filler, pore size, micromechanical interlocking, physicochemical properties

## Abstract

The filler/resin matrix interface interaction plays a vital role in the properties of dental resin composites (DRCs). Porous particles are promising fillers due to their potential in constructing micromechanical interlocking at filler/resin matrix interfaces, therefore improving the properties of the resulting DRCs, where the pore size is significantly important. However, how to control the pore size of porous particles via a simple synthesis method is still a challenge, and how their pore sizes affect the properties of resulting DRCs has not been studied. In this study, porous silica (DPS) with a dendritic structure and an adjustable pore size was synthesized by changing the amounts of catalyst in the initial microemulsion. These synthesized DPS particles were directly used as unimodal fillers and mixed with a resin matrix to formulate DRCs. The results showed that the DPS pore size affects the properties of DRCs, especially the mechanical property. Among various DPS particles with different pore sizes, DPS6 resulted in 19.5% and 31.4% improvement in flexural strength, and 24.4% and 30.7% enhancement in compression strength, respectively, compared to DPS1 and DPS9. These DPS particles could help to design novel dental restorative materials and have promising applications in biomedicine, catalysis, and adsorption.

## 1. Introduction

Dental caries is one of the most common diseases in the oral cavity [1]. The appropriate selection of restorative materials is essential for caries treatment [2]. Dental resin composites (DRCs), which are mainly composed of an inorganic enhancing phase and an organic continuous phase, are the most popular restorative materials for caries treatment due to their superior performances and operability [3,4]. However, their average life span is less than 10 years, and restoration fracture is one of the major causes [5,6]. 

Numerous studies have confirmed that restoration fracture is mainly caused by poor filler/resin matrix interfacial interaction [7,8,9], which is affected by the filler surface properties and morphology structure [10,11]. Generally, interfacial bonding is achieved by chemically silanizing the filler surface [12]. However, ester bonds and silanol could be hydrolyzed in the oral environment during the service, leading to inferior physiochemical performance [13]. In comparison, fillers with porous structures aid in physically constructing micromechanical interlocking with the resin matrix at the interface [14,15], which can improve the interfacial bonding through physical chimerism and is beneficial for enhancing the properties of the composite [16]. Based on the chimerism principle, micromechanical interlocking is mainly achieved by penetrating resin monomers into the pore channels of porous particles; thus, the pore structure/morphology/size characteristics of porous fillers significantly affect the interfacial interaction [4,17,18]. Our previous study demonstrated that dendritic porous silica particles (DPSs) with a hierarchical pore structure are beneficial for micromechanical interlocking with the resin matrix, further enhancing composite properties due to the increased fillers/resin matrix interfacial contact area [4]. However, how their pore sizes affect the construction of micromechanical interlocking at the filler/resin matrix interface has been rarely studied. The relationship between the filler pore size and the resulting composite properties is still unknown. 

In this study, we synthesize DPS particles with a hierarchical pore structure using a facile and simple method, and their pore size can be precisely controlled by changing the amounts of catalyst (HMT) in the initial microemulsion. To the best of our knowledge, there are few reports on the facile synthesis of these DPS particles with controllable pore sizes by only adjusting one parameter. Then, these DPS particles are characterized and directly used as the unimodal fillers for bisphenol A diglycidyl ether dimethacrylate (Bis-GMA)/triethylene glycol dimethacrylate (TEGDMA)-based dental composites, and the comprehensive properties of the resulting DRCs are evaluated. The aims of this study are to synthesize pore-size-tunable DPS particles and investigate the effects of their pore size on the properties of DPS-reinforced DRCs. The null hypothesis was as follows: the DPS pore size did not influence the physicochemical and mechanical properties of DPS-reinforced RBCs.

## 2. Materials and Methods

### 2.1. Materials 

Hexamethylenetetramine (HMT), Cetyltrimethylammonium bromide (CTAB), tetraethyl orthosilicate (TEOS), cyclohexane and ethanol were purchased from Sinopharm Chemical Reagent Co., Ltd. (Shanghai, China). N-pentanol, resin monomer (bisphenol A glycer-olate dimethacrylate (Bis-GMA) and tri(ethyleneglycol) dimethacrylate (TEGDMA)) and photoinitiator (camphorquinone (CQ) and ethyl-4-dimethylaminobenzoate (4-EDMAB)) were procured from Sigma-Aldrich. The human dental pulp cells (hDPCs) were obtained from dental pulp tissue provided by Shanghai Ninth People’s Hospital (Shanghai, China), based on the informed consent of patients. Dulbecco’s modified eagle’s medium (DMEM), fetal bovine serum (FBS) and trypsin were purchased from Gibco. Penicillin-streptomycin-amphotericin B solution, phosphatic buffer solution (PBS), Calcin-AM/PI Double Staining Kit and Cell Counting Kit-8 (CCK-8) were purchased from Beyotime. 

### 2.2. Synthesis of DPS Particles

Based on our previous work [4], DPS particles with different pore sizes were synthesized via a combined dynamic self-assembly and calcination process using CTAB as a template, TEOS as a silica source and HMT as a catalyst. Their pore sizes could be precisely controlled by regulating the amount of HMT. Briefly, 1.8 g CTAB and HMT with various masses (0.1, 0.3, 0.6 and 0.9 g) were added to 30 mL deionized water and stirred for 5 min at room temperature (RT; 25 °C). Then, 1.5 mL N-pentanol and 2.5 mL TEOS were added to the above solution and stirred for 30 min. Subsequently, the reaction mixture was transferred into a Teflon-lined autoclave and heated at 130 °C for 4.5 h. After natural cooling to RT, the synthesized particles were collected via centrifugation, dried in a vacuum oven at 90 °C and calcined at 550 °C for 6 h. The obtained powders were denoted as DPS1, DPS3, DPS6 and DPS9 with different amounts of HMT. 

### 2.3. Fabrication of DPS-Reinforced DRCs 

First, the resin matrix was obtained by thoroughly mixing Bis-GMA (49.5 g), TEGDMA (49.5 g), 4-EDMAB (0.8 g) and CQ (0.2 g) at RT, and it was collected in the dark for further fabrication. Second, the synthesized DPS particles were used as unimodal fillers and mixed with the above resin matrix to fabricate DRCs using a constant filler loading of 21 wt.%. Specifically, DPS particles were premixed with the resin matrix using a speed mixer (DAC 150.1 FVZ-K, FlackTek, Inc., SpeedMixer, Landrum, SC, USA). The mixture was then thoroughly blended using a three-roller mixer (EXAKT 80E, Norderstedt, Germany) to obtain the composite paste. Finally, the prepared composite pastes were de-bubbled in a vacuum oven at 25 °C for 12 h. To evaluate the physicochemical properties of the obtained DRCs, the uncured composite paste was applied into appropriately sized silicon rubber molds and photo-cured using a polywave LED-LCU (Bluephase N-LED, Schaan, Principality of Liechtenstein) with a mean irradiance of 1200 mW/cm^2^. 

### 2.4. Characterization 

#### 2.4.1. Morphology and Structure of DPS Particles 

The morphology and pore sizes of DPS particles were observed via field emission scanning electron microscopy (FE-SEM; SU8010, Tokyo, Japan) and transmission electron microscopy (TEM; Talos F200S, Waltham, MA, USA). The crystalline structure, porous structure and specific surface area were analyzed and evaluated using X-ray diffraction (XRD; D/max-2550VB+/PC, Rigaku, Japan), small-angle X-ray scattering (SAXSess mc2, Graz, Austria) and N_2_ adsorption/desorption isotherms (Quadrasorb-SI, Pittsburgh, PA, USA), respectively. 

#### 2.4.2. Physicochemical Properties of DPS-Reinforced DRCs

(1) Degree of conversion (DC) and depth of cure 

Real-time DC was conducted using a Fourier transform infrared (FT-IR) spectrometer (Nicolet 8700, Waltham, MA, USA) equipped with an attenuated total reflection (ATR) crystal. A background reading was collected between 400 cm^−1^ and 4000 cm^−1^ using 32 scans at a resolution of 4 cm^−1^. Composite paste was directly placed on the top of the ATR crystal. The spectrum of the uncured composite was collected. Photo-curing was then applied at zero distance from the top surface for 60 s (*n* = 3). Then, the spectrophotometer screw was applied to fix the cured specimen tightly on the reading crystal. The spectral region between 1600 and 1700 cm^−1^ was selected to identify the heights of the aliphatic C=C absorbance peak at 1638 cm^−1^ and the aromatic C=C absorbance peak at 1608 cm^−1^. The DC was calculated using the following Equation [19]:

$$DC(\%)=\left[1-\frac{(h_{1638}/h_{1608}{)}_{cured}}{{\left(h_{1638}/h_{1608}\right)}_{uncured}}\right]\times 100\%\;$$

where *h*_1638_ and *h*_1608_ are the height of aliphatic C=C peak and aromatic C=C peak, respectively.

The depth of cure of the DRCs was evaluated by placing the composite paste into a cylindrical mold (Φ 4 mm × 10 mm) and vertically irradiating it for 20 s (*n* = 3). After the specimen was de-molded and the excess paste was removed, the specimen height was measured at three different positions using a digital calliper (DL91150, Shanghai, China). The mean values divided by two were recorded as the depth of cure result, according to the American National Standards Institute (ANSI)/American Dental Association (ADA) specification NO. 27-2009 (ISO-4049) [20]. 

(2) Viscosity

The viscosity of uncured DRCs was measured via a rheometer (Thermo HAAKE MARS 60, Waltham, MA, USA) using a parallel-plate mode (20 mm) at 37 °C (*n* = 3). The plate gap was 1 mm, and the angular frequency range was 0.1–100 rad/s. 

(3) Mechanical properties

The mechanical properties of the formulated DRCs, including flexural strength (FS), flexural modulus (FM), and compressive strength (CS), were measured using a universal testing machine (Instron 5969, Norwood, MA, USA). The load was applied at a cross-head speed of 0.75 mm/min, in accordance with our previous work [4]. For FS and FM measurements, the composite pastes were applied to a rectangular-shaped mold (25 mm × 2 mm × 2 mm) and photo-cured using LED-LCU for 60 s on each side (*n* = 6). The cured specimens were polished with 1500-grit silicon carbide paper. Three-point bending test was applied according to ISO 4049-2009 [20]. Similarly, the cylindrical specimens (Φ 4 mm × 6 mm) were prepared and measured to obtain CS values (*n* = 6). 

(4) Fracture morphology 

The fracture morphologies of the specimens after the three-bending tests were further analyzed via FE-SEM. 

(5) Wear resistance 

Three specimens of each DRC (Φ10 mm × 1 mm) were prepared for wear resistance measurement. The specimens were photo-cured for 60 s on each side. After 24 h of dry storage at 37 °C, the top surfaces of the specimens were polished using a polishing machine (BETA-VECTOR, Buehler Co., Lake Bluff, IL, USA) with P1200 silicon carbide (SiC) paper under water cooling with an interval of 10 s. Then, the specimens were cleaned using an ultrasound water bath (Elma ultrasonic T 310, Singen, Germany) for 3 min. 

Surface roughness before and after 10, 20 and 30 s polishing were determined via Mahr Perthometer M1 (Lt = 5.6 mm, λc = 0.800 mm). Ra, which is the arithmetic mean of the sum of roughness, was recorded. Three vertical and three horizontal lines were measured on the specimen surface, and the mean Ra result was calculated. The morphology of the specimens after 30 s of polishing was further evaluated via FE-SEM. 

(6) Cytotoxicity assessment

We first extracted and collected human dental pulp cells (hDPCs), and the process is briefly described as follows: The collected teeth from the patient were immediately cleaned with PBS, disinfected with alcohol and then mechanically split to obtain pulp tissue. Subsequently, the pulp tissue was quickly cut into small pieces and pressed with a slide cap in a Petri dish; then, the medium (DMEM containing 10% FBS and 1% Penicillin-streptomycin-amphotericin B solution) was added and placed in a cell incubator (5% CO_2_, 37 °C), where the medium was changed every 4 days. All of the operations were carried out in a sterile environment. After about 10 days, the hDPCs crawled out from the tissue mass, and the primary hDPCs could be obtained using trypsin. The primary hDPCs continued to be cultured and passed regularly, and they were collected for further use when passed on to the third generation. 

Meanwhile, the photo-cured specimens (Φ 10 mm × 1 mm, *n* = 3) were prepared. Both sides of the specimens were wiped with 75% alcohol, underwent ultraviolet (UV) light irradiation for 30 min, and then cleaned with phosphate-buffered saline (PBS) 3 times. Then, the specimens were incubated using DMEM containing 10% FBS and 1% penicillin-streptomycin-amphotericin B solution in a 12-well tissue culture plate for 24 h (5% CO_2_, 37 °C). The specimen extracts were obtained. Based on ISO 10993-5 [21], the culture medium volume to the surface area of the specimen is 1 mL: 1.25 cm^2^. The human dental pulp cell (hDPCs) suspension (6000 cells/mL) was seeded into 96-well plates (100 μL/plate) and incubated for 24 h (5% CO_2_, 37 °C). The culture media were replaced with the specimen extracts and cultured for 1, 3, 5 and 7 days. During each culture period, the viability levels of the hDPCs were assessed using a CCK-8 assay (λ = 450 nm), and the optical density (OD) was evaluated using a microplate reader (Elx 800, Winooski, VT, USA). The cell morphology was evaluated by a LIVE/DEAD cell imaging kit (calcein-AM/propidium iodide (PI)) and a fluorescence microscope (Leica DMi8, Weztlar, Germany) in each culture period. 

### 2.5. Statistical Analysis 

All statistical analyses were analyzed using one-way ANOVA and Tukey post hoc tests (*p* < 0.05). 

## 3. Results

### 3.1. Morphology Characterization of DPS Particles

**Figure 1:**
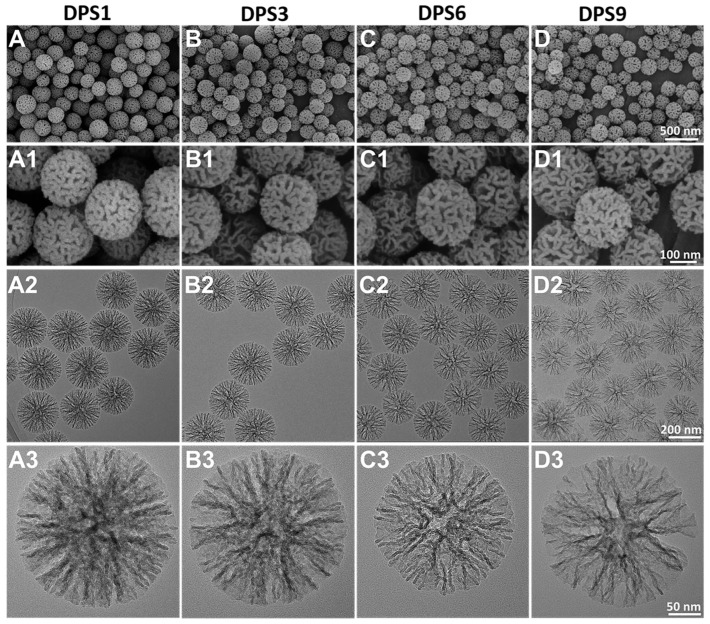
FE-SEM (**A**–**D1**) and TEM images (**A2**–**D3**) of DPS particles with different magnifications: DPS1 (**A**–**A3**), DPS3 (**B**–**B3**), DPS6 (**C**–**C3**) and DPS9 (**D**–**D3**).

The FE-SEM and TEM images indicate that the monodisperse spherical DPS particles present a hierarchical pore structure with a highly uniform particle size (~240 nm) (Figure 1), and the pore channels of these particles radiate from the center in a three-dimensional distribution manner (Figure 1A2–D3).

### 3.2. Structure Characterization of DPS Particles

**Figure 2:**
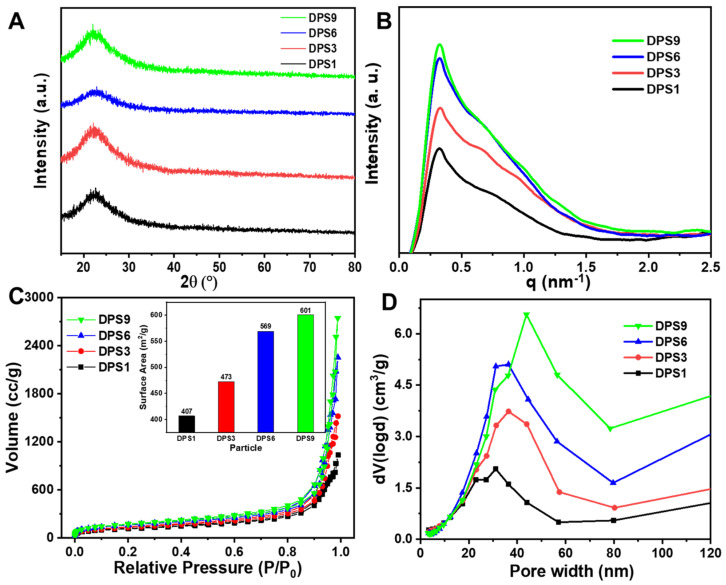
X−ray diffraction patterns (**A**), small angle X−ray scattering patterns (**B**), N2 adsorption−desorption isotherms (**C**) and pore size distributions (**D**) of DPS particles. The inset figure in (**C**) represents the surface areas of DPS particles.

The XRD results in Figure 2A reveal a broadened peak belonging to the amorphous silica according to the JCPDS 29-0085 [22]. In Figure 2B, the SAXS curves showed more than one scattering peak, indicating the presence of a hierarchical porous structure [23]. To further evaluate the structure and pore structure of DPS particles, the N_2_ adsorption/desorption was measured. In Figure 2C, the N_2_ adsorption/desorption curves presented a similar-type IV isotherm with an H3-hysteresis loop in 0.4 < P/P_0_ < 1.0 and all of the isotherms did not level off even at P/P_0_ = 1, indicating the presence of mesopores and macropores with various sizes in the materials [23,24,25]. Moreover, the pore size distribution patterns calculated using the density functional theory (DFT) method show that all of the DPS particles present multi-peaks (Figure 2D), further demonstrating the multimodal pore size distributions [4,26]. In addition, the specific surface area and cumulative pore volume of the DPS particles are proportional to the pore size, where the DPS1 has the lowest value, as shown in the inset figure in Figure 2C. The value of total pore volume at P/P_0_ = 0.99 is 1.602, 2.352, 3.489 and 4.246 cc/g for DPS1, DPS3, DPS6 and DPS9 particles, respectively. These results are consistent with the above FE-SEM and TEM results.

### 3.3. Physicochemical−Cytocompatibility Properties of DPS-Reinforced RBCs

#### 3.3.1. Degree of Conversion, Depth of Cure and Viscosity

**Figure 3:**
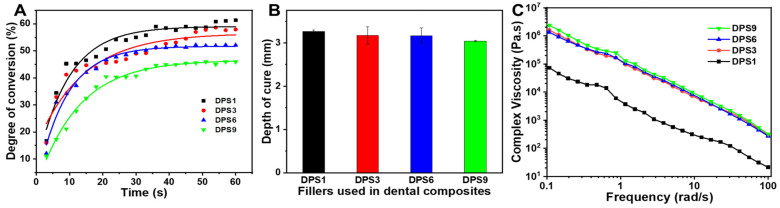
Degree of conversion (**A**), depth of cure (**B**) and complex viscosities (**C**) of the DRCs reinforced with DPS particles at 21 wt.% filler loading.

The real-time DC curves of the tested DPS-reinforced DRCs increase similarly (Figure 3A), and DPS9-reinforced DRCs present the lowest DC value. Meanwhile, the depth of cure results from the studied composites correspond to the DC results, and all of them meet the requirement of the ISO standard (>1.5 mm) [20], demonstrating that DPS particles with different pore sizes do not influence the DRC depth of the cure results. The rheology characteristic of DRCs is essential for their clinical applications [27]. In this study, all of the DRCs exhibited a non-Newtonian shear-thinning phenomena (Figure 3C), and DPS1-reinforced DRCs had the lowest viscosities. The result indicates that the pore sizes of porous fillers affect the composite viscosities.

#### 3.3.2. Mechanical Properties

In the present study, to study the effects of DPS particle pore sizes on the material mechanical properties, all DRCs with fixed 21 wt.% filler loading were fabricated. The filler loading value was determined according to the DPS9-reinforced DRCs due to the DPS9 particle having the highest specific surface area (Figure 2C).

**Figure 4:**
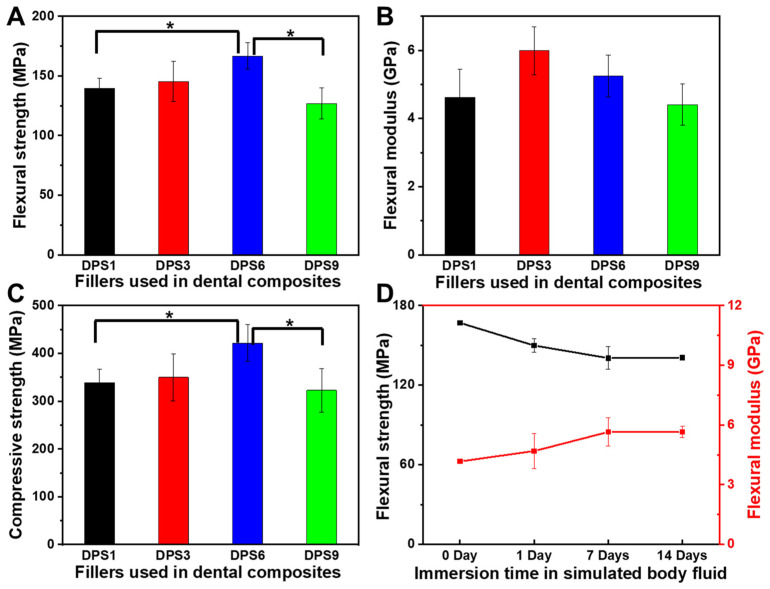
Flexural strength (**A**), flexural modulus (**B**) and compressive strength (**C**) of DPS-reinforced DRCs without immersion, and flexural strength and flexural modulus of DPS6-reinforced DRCs before and after immersion in simulated body fluid at 37 °C (**D**). The “*” indicates *p* < 0.05.

The FS, FM and CS values of DPS-reinforced DRCs were measured, and the results are presented in Figure 4. As expected, the pore sizes of the DPS particles directly affect the mechanical properties. As the pore size increases, the FS and CS values first increase and then decrease. Relative to DPS1 and DPS9, DPS6 resulted in 19.5% and 31.4% improvement in FS and 24.4% and 30.7% enhancement in CS, respectively (Figure 4A,C). The FM does not vary with the tested DRCs. DPS3- and DPS6-reinforced DRCs have relatively high FM values (Figure 4B).

To further evaluate the mechanical stability, the DPS6-reinforced DRCs with good FS and CS results are chosen to compare the changes in the FS and FM before and after being soaked in the simulated body fluid (SBF) for 1, 7 and 14 days (Figure 4D). FS decreases by 10.2% after 1 day, which may occur due to the plasticizing effects of the absorbed water [17,28]. However, there is no significant reduction in FS after 7 and 14 days, confirming the superior FS stability of the porous filler-reinforced DRCs [4]. The FM values do not change with soaking time, which can be explained by the fact that the modulus of the composites mainly depends on the filler fraction [29].

#### 3.3.3. Fracture Morphology

**Figure 5:**
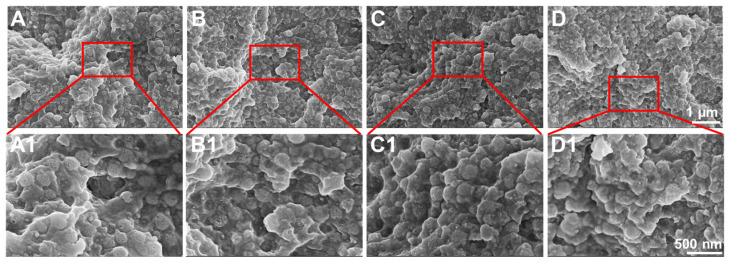
FE-SEM images of the fracture morphologies of DPS-reinforced DRCs: DPS1 (**A**,**A1**), DPS3 (**B**,**B1**), DPS6 (**C**,**C1**) and DPS9 (**D**,**D1**) with different magnifications.

For DRCs, the filler distributions in the resin matrix and the filler/resin matrix interfacial interaction are important for various properties of the resulting composites. In this study, the fracture morphologies of DPS-reinforced DRCs were analyzed. As shown in Figure 5, all of the DPS particles are uniformly dispersed in the resin matrix without agglomeration. The DPS1 particles are exposed and their porous structures can be clearly observed (Figure 5A1), indicating the poor adhesion of the resin matrix. In comparison, DPS6 and DPS9 particles with large pore sizes are embedded in the resin matrix, and their pore structures are barely observed (Figure 5C1,D1), indicating adequate interfacial bonding between the resin matrix and particles. Furthermore, the rough fracture surface of the DPS6-reinforced DRCs has more steps and cracks than the others (Figure 5C,C1), denoting its improved filler/resin matrix interfacial bonding [30].

#### 3.3.4. Wear Resistance

**Figure 6:**
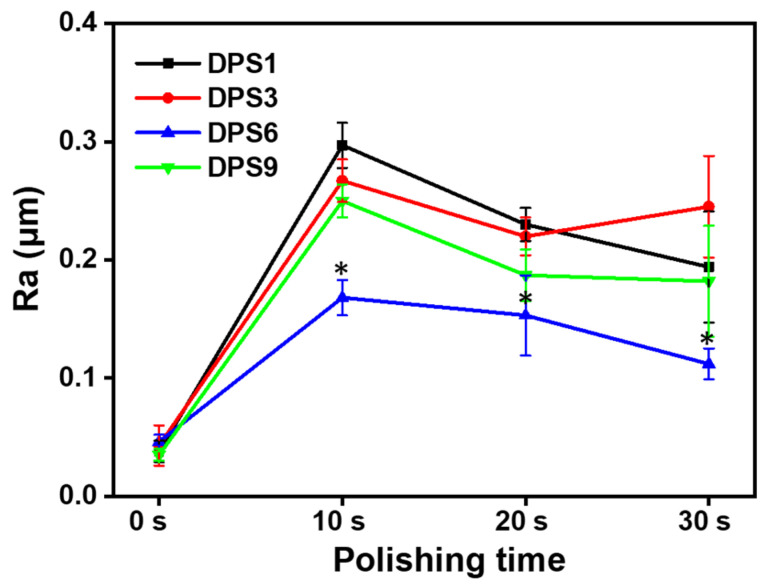
Surface roughness (Ra) of DPS-reinforced DRCs after 30 s polishing. The “*” indicates *p* < 0.05, compared with DPS6-filled DRCs.

**Figure 7:**
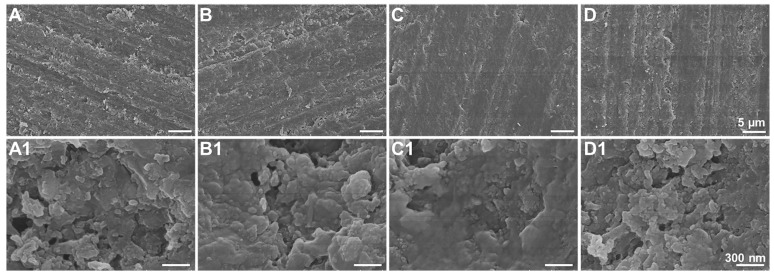
FE-SEM images of DPS-reinforced DRCs after 30 s polishing: DPS1 (**A**,**A1**), DPS3 (**B**,**B1**), DPS6 (**C**,**C1**) and DPS9 (**D**,**D1**) with different magnifications.

The surface roughness of the DPS-reinforced DRCs after 10, 20 and 30 s of polishing was evaluated (Figure 6), whereby DPS6 had improved wear resistance with Ra < 0.2 μm, which is the clinical threshold for bacterial retention. The FE-SEM images (Figure 7) demonstrate the surface roughness results and show that DSP6 has a smoother surface after polishing than the other specimens with obvious wear tracks. 

#### 3.3.5. Cytotoxicity Assessment

**Figure 8:**
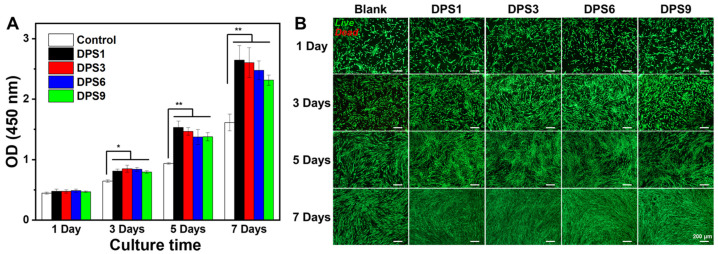
CCK-8 assay (**A**) and fluorescence images of LIVE/DEAD staining (**B**) of the DPS-filled dental composites after culturing for 1, 3, 5 and 7 days. The “*” indicates *p* < 0.05 and “**” indicates *p* < 0.01. The green represents living cells and the red represents dead cells.

The cell viability levels of DPS-reinforced DRCs were assessed using hDPCs. The OD values and LIVE/DEAD staining images are shown in Figure 8. The OD values of hDPCs were confirmed via CCK-8 assays (Figure 8A). These results show that the OD values of the studied DRCs gradually increase from days 1 to 7. In addition, DPS-reinforced DRCs exhibit higher cell viability at each time point than the control group; additionally, there are no statistically significant differences among the studied DRCs, indicating their improved cytocompatibility levels. 

The morphology of hDPCs cultured in different composite extracts was further assessed via the LIVE/DEAD assay (Figure 8B). The numbers of live hDPCs gradually increase with the extension of the culturing time, and few dead cells can be observed in each culture stage, especially for DPS-reinforced DRCs, demonstrating their excellent cytocompatibility. This result is consistent with the above CCK-8 results. 

## 4. Discussion

All of the DPS particles were monodisperse and spherical in shape with a highly uniform particle size. In addition, the pore size of the DPS particles can be regulated by changing the amount of HMT in the initial microemulsion reaction system, in which the DPS1 and DPS9 possess the smallest and largest pore size, respectively (Figure 1A3–D3). This result demonstrates that the catalyst plays an important role in adjusting the pore size of the DPS particles, and the specific influencing mechanism will be studied in our further work. The structure and pore size changes in the DPS particles were further demonstrated via SAXS and pore size distribution curves, and the results confirmed their multimodal pore size distributions. 

Our previous study demonstrated that the porous particles were promising fillers due to their potential in constructing micromechanical interlocking at filler/resin matrix interfaces. Based on the principle of mechanical chimerism between the porous fillers and resin matrix, the pore size should be important to fabricate the excellent filler/resin matrix interface interaction. Hence, in this study, the DPS particles with a hierarchical pore structure and adjustable pore sizes were used as unimodal filler in the Bis-GMA/TEGDMA resin matrix to fabricate DRCs, and the comprehensive properties of DRCs were studied.

The degree of conversion (DC) is a critical characteristic for determining the corresponding properties of dental composites. DC rarely reaches 100% because of the trapped radicals in the cross-linked networks [31]. The unreacted monomers would produce a plasticizing effect, degrading the mechanical properties of the corresponding DRCs. In addition, the free monomer can result in DRC cytotoxicity, which is not conducive to clinical use [32]. The DC values are mainly affected by various inherent factors, including the resin matrix composition [33,34], filler loading [35,36] and filler structure [37,38]. In this study, the DC and the depth of cure of all of the DPS-reinforced BRCs showed no significant differences, which may be attributed to the similar DPS particle structures and fixed filler loadings. In comparison, the final DC values of the studied DRCs are lower than those of nonporous spherical SiO_2_ particle-reinforced DRCs with the same filler loadings. The result may be related to the refracted light via the DPS particle pore structure during light-curing [4], potentially weakening the light intensity and affecting the photo-polymerization of the resin matrix into the pore channel. The above phenomenon merits further investigation.

As the shear frequency increases, the viscosity of the studied DRCs decreases, which is suitable for clinical applications. These DRCs can readily flow and adapt to the cavity under an exerting force [39]. Among all of the samples, DPS1-reinforced DRCs have the lowest viscosities, which is mainly due to the small pore size and the relatively small surface area of the fillers. The result indicates that the pore sizes of porous fillers affect the composite viscosities. Among all of the studied DRCs in this study, DPS6 presented the best enhancement effect. This result can be explained by the fact that the resin matrix may not be able to enter into the DPS1 smaller pore channel, and the empty channel exists in DPS9-reinforced DRCs due to the resin matrix not being able to completely occupy the DPS9 larger pore channel, whereby all of which will lead to small contact areas between the fillers and resin matrix and a weak micromechanical interlocking structure. Therefore, the filler particles are easy to remove from the resin matrix under the external force, resulting in inferior FS and CS results. The relatively high FM values of DPS3- and DPS6-reinforced DRCs may be related to their appropriate pore sizes and the resulting sufficient interfacial bonding strengths. After applying the stress, particles are difficult to be pulled out of the resin matrix, increasing the resistance against bending deformation. Due to the sufficient infiltrated resin matrix within the porous channel of the filler, good resin matrix/filler interfacial bonding leads to superior material mechanical properties. All of the fracture morphology results support the mechanical property findings (Figure 4). The wear resistance levels of DRCs mainly depend on filler properties and the resin matrix/filler interfacial bonding strengths [40]. Some studies have reported that composites reinforced by porous fillers have good wear resistance since the strong micromechanical interlocking between the resin matrix and filler prevents filler detachment [15,41,42]. Thus, the improved filler/resin matrix interfacial bonding of the DPS6-reinforced DRCs prevents the particles from being removed from the resin matrix during wear. However, the depth of the resin matrix penetrating the filler pore structure merits further investigation. In addition, based on the cytotoxicity assessment with CCK-8 and LIVE/DEAD staining images, all of the DPS-reinforced RBCs presented good cytocompatibility. All of the results demonstrate that the pore size of DPS particles affects the properties of the corresponding DRCs. Thus, the null hypothesis was rejected. The pore size of DPS particles affects the properties of reinforced DRCs.

## 5. Conclusions

DPS particles with uniform particle size distribution and a dendritic hierarchical porous structure were successfully synthesized via a dynamic self-assembly calcination process. The DPS pore size could be simply tuned by changing one parameter (the amount of catalyst) in the initial microemulsion reaction system. The DPS particles were used as fillers in DRCs, and the results demonstrated that their pore size affected the properties of corresponding DRCs. The result from this study provided a comprehensive understanding of how the filler pore size affects the physicochemical–cytocompatibility properties of DRCs, which is beneficial for regulating composite properties. DPS particles are promising inorganic-based reinforced fillers in DRCs, but their filler loading is much lower than commercial DRCs. Hence, further studies on optimizing the filler formulations of the DPS-based reinforced DRCs, including filler loading and compositions, evaluating the comprehensive physicochemical performances and comparing these results to DRCs on the market will make us further deepen the understanding of this unique filler.

## Data Availability

The datasets used and analyzed during the present study are available from the corresponding author upon reasonable request.
